# Multi-informant path models of the influence of psychosocial and treatment-related variables on adherence and metabolic control in adolescents with type 1 diabetes mellitus

**DOI:** 10.1371/journal.pone.0204176

**Published:** 2018-09-20

**Authors:** Lene Juel Kristensen, Niels Holtum Birkebaek, Anne Hvarregaard Mose, Morten Berg Jensen, Mikael Thastum

**Affiliations:** 1 Department of Psychology and Behavioural Sciences, Aarhus University, Aarhus, Denmark; 2 Department of Pediatrics, Aarhus University Hospital, Aarhus N., Denmark; 3 Department of Economics and Business Economics, Aarhus University, Aarhus, Denmark; McMaster University, CANADA

## Abstract

**Background:**

We assessed the associations between metabolic control and adherence and a broad range of adolescent and family characteristics (e.g., gender, family structure), treatment-related variables (e.g., disease duration, treatment modality), and psychosocial factors (e.g., symptoms of depression and anxiety, parental support, self-efficacy) in a nationwide study of Danish adolescents (age 12–17 years) with type 1 diabetes mellitus (T1DM).

**Methods:**

Sixty-four percent of invited families participated by completing a survey and providing a blood sample. Two path models of associations between generic and diabetes-related family factors, adolescent self-efficacy, emotional difficulties, and metabolic control and adherence were tested, one for adolescents and one for caregivers. Demographic variables were included as covariates.

**Results:**

Both path models demonstrated a satisfying model fit. In both models, metabolic control was associated with adherence, age, and T1DM duration. In the adolescent model, metabolic control was also related to treatment modality, single-parent household, caregiver non-support, and anxiety, whereas in the caregiver model metabolic control was associated with family conflict and caregiver support. In both models, adherence was related to age, duration, treatment modality, family conflict, caregiver support, family functioning, and emotional difficulties of the adolescent. In the adolescent model, adherence was also related to adolescent self-efficacy, whereas in the caregiver model adherence was associated with adolescent gender and caregiver non-support and support. Adolescent self-efficacy, emotional well-being, and difficulties related to adolescent/caregiver interaction appeared to be particularly important, as indicated by their stronger association with adherence and/or metabolic control.

**Conclusion:**

The results highlight the value of applying a multi-informant approach to address the psychosocial well-being of adolescents with diabetes in a large national sample. Self-efficacy, emotional, and family-related difficulties are important aspects to address in both clinical care and future research regarding adolescents with T1DM.

## Introduction

Achieving adequate metabolic control is crucial in children and adolescents with type 1 diabetes mellitus (T1DM) to prevent both immediate and long-term health complications [1, 2]. The many tasks necessary to achieve successful diabetes management is perceived by many patients and caregivers as a huge burden, with adherence to diabetes treatment a necessary, but not always sufficient, prerequisite for optimal control [3]. Biological, physiological, and psychosocial factors act as intricate parts of the complex processes that influence metabolic control and adherence in adolescents with T1DM.

A range of variables related to individual and family characteristics have been shown to affect T1DM treatment. Older age, longer disease duration, and being treated with multiple daily injections (MDI) vs. insulin pump/continuous subcutaneous insulin infusion (CSII), living in a single-parent household, lower education level of parents, and lower family income have all been associated with decreased adherence and worsening metabolic control [4–7]. However, any association of gender with either metabolic control or adherence is unclear [8–10].

The prevalence of problematic psychological responses by adolescents with T1DM, such as symptoms of depression and anxiety, differs greatly among studies [11–14] but, regardless of the overall prevalence, the presence of these symptoms should warrant attention. Some studies have shown an association between deteriorating metabolic control and mental health problems [15, 16], and adherence has been associated with depression [17, 18] and anxiety [16]. Other studies have not been able to confirm these associations [19].

Previous research has consistently found adolescent self-efficacy (defined as the belief that one can carry out specific behaviors in specified situations [20]) in relation to diabetes management to be an important factor in diabetes care and to be associated not only with adherence, but also metabolic control [21–23], possibly with self-efficacy acting as a mediator between diabetes-specific family issues (e.g., diabetes-related conflict, non-support, or diabetes responsibility) and adherence or metabolic control [24, 25]. A number of studies have focused on conflict and arguments in the family related to diabetes-specific tasks and behaviors and found a direct association with poorer metabolic control [26–28]. Other studies have found an association between conflict and metabolic control to be mediated by adherence [29] or found no association between diabetes-related family conflict and adherence [30].

Both parental support and non-support (as evidenced by critical and negative parenting) in relation to diabetes-specific tasks and behaviors have been shown to be associated with both adherence and metabolic control [31, 32], possibly with adherence mediating the association between critical parenting and glycosylated hemoglobin (HbA_1c_) as the prevailing indicator of metabolic control [33]. However, further investigations of the link between support, non-support, adherence, and metabolic control is needed due to the so far inconsistent results.

Parental involvement in relation to responsibility in diabetes management tasks has been associated with adherence, and possibly metabolic control through the mediational effect of adherence [34, 35]. A previous study found that adolescents who perceive greater caregiver responsibility engage in better diabetes management, as measured by blood glucose monitoring [36]. Aspects of the family’s general way of functioning, such as family cohesion and family dysfunction, have been associated with adherence and metabolic control in some studies [37, 38], whereas other studies have found no relationship with metabolic control [39].

Many of the abovementioned studies relied on relatively small, homogenous groups of participants, and larger studies that are able to test comprehensive models reflecting the multifaceted complexity of factors influencing daily diabetes management, and thereby metabolic control, are needed. Models including a larger number of variables, while still seeking to describe the relative and unique contribution of each, are necessary to guide the multidisciplinary clinical care of adolescents with T1DM and the development of effective screening and interventions. Whittemore, Jaser, Guo and Grey [40] have proposed a theoretical model based on results of previous studies that encompass the possible associations of a multitude of variables relating to individual and family characteristics, psychosocial, individual, and family responses, with adaptation outcomes such as metabolic control. This model is intended as a conceptual framework to guide researchers and health care providers in their understanding of the complexity of diabetes treatment and adaption to living with this chronic disease. Whittemore et al. stresses the importance of conducting further research to confirm and develop the model and the inconsistent results on which some of the presumed association are based. This model has inspired the development of the path models being tested in the current study.

Thus, the present study set out to investigate possible associations between adherence or metabolic control and patient characteristics, treatment aspects, and psychosocial and psychological variables in Danish children and adolescents (age 12–17 years) with T1DM. As previous research has highlighted the often differing perspectives of adolescents and caregivers in assessing health and the family milieu, we tested separate, independent multivariate path models for children/adolescents and caregivers in order to examine possible direct and indirect pathways between a broad range of variables and metabolic control and adherence. Based on previous research, poorer metabolic control and lower levels of adherence were expected to be independently associated with more diabetes-related family conflict, more parental non-support, and less parental support. These outcomes were also expected to be related to less adolescent self-efficacy in relation to diabetes care and more symptoms of depression and anxiety, and family division of responsibility and general family functioning were expected to be related to adherence, which would act as a mediator between metabolic control and emotional and social difficulties, diabetes-related family conflict, parental support, and non-support. Furthermore, the possible influence of age, gender, diabetes duration, treatment modality, and caregiver socioeconomic factors in relation to both adherence and metabolic control was assessed.

## Materials and methods

### Ethics statement

The department where the study was conducted did not have an institutional review board, as this is not standard in Denmark. Thus, in accordance with Danish procedure, the regional ethic committees (De Videnskabsetiske Komitéer for Region Midtjylland) was consulted. In keeping with the regulations of the committees, questionnaire-based studies do not require permission prior to initiation; however, a study protocol was provided to the committees who confirmed, that although participating children and adolescents were asked to submit a small blood sample (comparable to their daily blood glucose testings), no biological samples were collected with the intent of establishing a research bio-bank, therefore the project was not encompassed by the term ‘Bio-medical research’, and as such not eligible for Committee review and approval. The project was registered with the Danish Data Protection Agency (Ref no. 2013-41-1528). Written informed consent was obtained from all participants or their caregivers through the online version of the questionnaires or on paper. All families were given thorough written information informing them that their participation was voluntary and anonymous to everyone other than the first author, and that their consent could be withdrawn at any time, just as refusal to participate did not in any way influence the diabetes treatment that the child/adolescent was receiving.

### Participants and procedure

The present study was part of a nationwide web survey initiated to assess the influence of psychosocial variables on adherence, metabolic control, and quality of life in all Danish children and adolescents with T1DM (age 2–17 years). The study was conducted in collaboration with the Danish Society for Diabetes in Childhood and Adolescence, who administers the Danish Registry for Childhood and Adolescent Diabetes (DanDiabKids). Since 1996, DanDiabKids has collected data on all children and adolescents in Denmark with a diagnosis of T1DM, including annual registration of current HbA_1c_ levels, which are analyzed centrally to ensure conformity.

Based on information from DanDiabKids, all families in Denmark with a child or adolescent between 2 and 17 years of age with a diagnosis of T1DM (*n* = 1739) were invited to participate. We excluded 258 families who were registered as being unwilling to participate in scientific research, had a protected address, or were no longer residing at the address registered in the Danish Civil Registration System from which all participant addresses were collected. All families received a written invitation by post, asking them to participate in the national web survey. They were also given the option of completing a paper version of the questionnaire if they preferred.

The caregiver primarily involved with the diabetes-related care of the child/adolescent was requested to complete the survey. All families were asked to provide a blood sample from the child for HbA_1c_ analysis. A total of 1075 of the invited families had a child/adolescent (hereafter referred to as ‘adolescents’) with T1DM between 12 and 17 years of age. Based on their age, these adolescents were deemed mature enough to complete the full self-report questionnaire battery by themselves if desiring to do so. 519 of these adolescents, and 531 of the caregivers completed the required questionnaires, and consequently, data from these adolescents and one of their caregivers form the basis of the present study and analysis (see S1 Fig).

A paired samples t-test revealed no significant differences between the HbA_1c_ values provided for the study (M = 8.23, SD = 1.24) and those obtained from DanDiabKids for the same participants (M = 8.21, SD = 1.28, t (579) = 0.65, p = 0.52). The HbA_1c_ values from DanDiabKids were used for the analyses if participants did not provide a blood sample for the study.

Patient characteristics, including family structure and Danish as primary language in the home (a proxy marker for ethnicity), are summarized in Table 1.

**Table 1:** Participant characteristics.

	Percentage or mean (standard deviation)	Adolescent report	Parent report
Age of participating adolescents, years	14.6 (1.6)		
Gender of participating adolescents, girls	50,0%		
HbA1c - %	8.25 (1.25)		
HbA1c - mmol/mol	66.7 (13.7)		
Diabetes duration, years	6.07 (3.47)		
Participants using insulin pump	37.8%		
Gender of participating caregiver, female	83.6%		
Living with parents who are no longer together	20.8%		
No longer living with either parent (living with foster parents, at a continuation school, a treatment facility, or living by themselves)	5.2%		
Danish primary language at home	98.7%		
Household income:			
• Less than 400.000 kroner* • Between 400.000–499.999 kroner* • 500.000 kroner or more*	10.5% 12.8% 76.7%		
Adherence (ADQ)		3.95 (0.58)	3.96 (0.62)
Anxiety (BAI)		8.11 (7.78)	
Depression (BDI)		7.58 (8.85)	
Emotional and behavioral problems (SDQ)			7.48 (5.81)
Self-efficacy (SEDM)		7.19 (1.71)	
General family functioning (FAD)		20.70 (5.66)	20.61 (5.65)
Diabetes-related family conflict (DFCS)		20.57 (5.25)	20.46 (4.65)
Supportive caregiver behavior (DFBC sup)		26.35 (6.50)	27.94 (5.54)
Non-supportive caregiver behavior (DFBC non-sup)		14.62 (5.05)	14.38 (4.50)
Responsibility for diabetes-related tasks (DFRQ)		33.52 (4.82)	31.96 (5.03)

* Annual household income before taxes.

Independent samples t-tests revealed no significant difference between participants and non-participants regarding age (t (1165) = 1.22, p = 0.22), but non-participants had been diagnosed with T1DM for a slightly longer duration (M = 6.83, SD = 1.61, t (1160) = 3.71, p = 0.00) and were in worse metabolic control (M = 8.89, SD = 1.53, t (1153) = 7.29, p = 0.00). Of the participating adolescents, 26.7% met the recommended HbA_1c_ level (< 7.5%/58 mmol/mol).

### Measures

#### Treatment adherence

Adherence to diabetes treatment was assessed using the Adherence in Diabetes Questionnaire (ADQ), which was developed for this study. The psychometric properties of the questionnaire were described previously [41]. Caregivers and adolescents completed the 17 or 19 items (depending on treatment modality) of the ADQ assessing adherence to different aspects of diabetes treatment. The questionnaires were scored by calculating the mean of all items. Higher scores indicate better adherence. Within this study sample, the ADQ demonstrated acceptable internal consistency, with Cronbach’s alpha ranging from 0.89 (MDI version) to 0.85 (CSII version) on the caregivers’ responses, and 0.85 (MDI version) to 0.82 (CSII version) on the adolescents’ reports.

#### Supportive and non-supportive caregiver behavior

Parental support and non-support in relation to diabetes care were assessed using the Diabetes Family Behavior Checklist (DFBC) [42, 43]. Both caregivers and adolescents completed the questionnaire, which consists of two separate subscales: nine items comprising the support scale assessing both affective and practical support, and seven items comprising the non-support scale assessing diabetes-related critical parenting behavior. Higher scores on the support scale indicate the child’s or caregiver’s perception of more parental support in relation to diabetes, and higher scores on the non-support scale indicate a perception of more critical parenting/non-support. The reliability and internal consistency of the subscales of the DFBC were previously found to be adequate [31, 44]. In the present study, the Cronbach’s alpha was 0.69 (caregivers)/0.74 (adolescents) for the support subscale and 0.70 (caregivers)/0.64 (adolescents) for the non-support subscale.

#### Diabetes-related family conflict

The presence of diabetes-related family conflict was assessed using the Diabetes Family Conflict Scale (DFCS). Both adolescents and caregivers completed the revised version of the DFCS, which has demonstrated satisfactory internal consistency, adequate concurrent validity, and predictive validity in relation to metabolic control [26]. The DFCS is scored by calculating the sum score for all items, with higher scores indicating more conflict. The Cronbach’s alpha was 0.87.

#### Responsibility for diabetes-related tasks

Both caregivers and adolescents completed the Diabetes Family Responsibility Questionnaire (DFRQ) to assess the division of responsibility in relation to regimen tasks, general health maintenance, and social presentation of diabetes [45]. This instrument is widely used and has proven to be psychometrically sound [36]. In the present study, a total responsibility score was calculated by summing the item responses, with a higher score indicating more adolescent responsibility. The Cronbach’s alpha was 0.84 for the caregiver scale and 0.82 for the adolescent scale.

#### General family functioning

The General Functioning subscale of the Family Assessment Device (FAD) was chosen as a measure of generic family functioning. The FAD is based on the McMaster Model of Family Functioning [46] and comprises six subscales describing six dimensions of family functioning. The 12-item General Functioning subscale was completed by both caregivers and adolescents, with a higher score indicating unhealthier functioning. The psychometric properties of both the overall questionnaire and the General Functioning subscale were described previously and deemed satisfactory [46, 47], just as the FAD has been used extensively in pediatric samples [48].

The Cronbach’s alpha of the subscale in the present study was 0.89 for caregivers and 0.87 for adolescents.

#### Social and emotional difficulties of the adolescent

Adolescents completed the depression (BDI) and anxiety (BAI) subscales of the Beck’s Youth Inventories–second edition (BYI-II) [49]. Each subscale consists of 20 questions and is scored by calculating a total score ranging from 0 to 60, with higher scores indicating more symptoms of depression or anxiety. The reliability and test-retest stability of the Danish version of the BYI was found to be adequate [50]. In the present study, the Cronbach’s alpha was 0.94 for the BDI subscale and 0.92 for the BAI subscale.

The caregivers completed the Strength and Difficulties Questionnaire (SDQ). The SDQ is a brief, 25-item behavioral screening instrument consisting of five separate subscales that generate scores for Emotional Symptoms, Conduct Problems, Hyperactivity-Inattention, Peer Problems, and Prosocial Behavior [51]. Only the Total Difficulties Score calculated by summing the scores on the first four subscales was used in the present study. The SDQ has been used worldwide, with satisfactory psychometric properties, including reliability and validity, being established [52, 53]. In this study, the Cronbach’s alpha for the 20 items of the Total Difficulties Score was 0.84.

#### Self-efficacy

The adolescents’ self-efficacy in relation to managing diabetes-related tasks was measured using the Self-Efficacy for Diabetes Self-Management (SEDM). The 10-item questionnaire is scored by calculating the mean of all items. Higher scores indicate a more positive perception of self-efficacy. The SEDM was previously shown to be both valid and reliable [54], and in this study the Cronbach’s alpha was 0.89.

#### Medical and sociodemographic information

Information regarding diabetes duration was provided by DanDiabKids, whereas caregivers provided information regarding family structure, caregiver education level, household income, and the adolescent’s current diabetes treatment.

Analyzing the blood samples provided by participants, HbA_1c_ was used as an objective measure of metabolic control over the most recent 8–12 weeks. Blood samples were analyzed at a central laboratory using high-pressure liquid chromatography (Tosoh Bioscience, South San Francisco, CA, USA) and standardized according to the American National Glycohemoglobin Standardization Program (NGSP).

The web-based survey took approximately 45 minutes to complete depending on reading proficiency and computer skills.

### Statistical analysis

Descriptive analyses were performed in SPSS version 24 (SPSS Inc., 2016).

Bivariate correlations between observed measures, including demographic covariates and metabolic control, were examined. In line with Cohen [55], we considered a correlation of 0.5 as large, 0.3 as moderate, and 0.1 as small. Path modeling was performed using Mplus 7.0 software [56].

Mediation was assessed using bias-corrected bootstrapped confidence intervals for the indirect effects as advocated by Shrout and Bolger [57]. Thus, we followed recent recommendations and did not use significance of the total effect as a prerequisite for assessing mediation [57]. The hypothesized models consisted of 10 and 8 variables for adolescents and caregivers, respectively. The analyses were carried out separately for adolescents and caregivers.

Data were screened for outliers and assessed for normality. Diabetes-related family conflict and the social and emotional difficulties of the adolescent exhibited a pronounced pattern of non-normality (right-skewed); therefore, we carried out the analysis using the robust maximum likelihood estimator, providing standard errors and measures of model fit, which are robust to non-normality. In the path analysis missing data was handled via maximum-likelihood for the endogenous variables. For the exogenous variables list-wise deletion is utilized. The latter leads to a reduction in the sample size of slightly below 25% for both children and adults (see S1 Fig). The overall model fit was assessed using the chi-square value with p > 0.05, root mean square error of approximation (RMSEA) <0.05, and standardized root mean square residual (SRMR) <0.08, indicating an acceptable fit of the model. Furthermore, a comparative fit index (CFI) and Tucker-Lewis Index (TLI) in excess of 0.95 indicates an acceptable fit of the model [58].

## Results

### Bivariate correlations

#### Correlations between study variables

Bivariate correlations between all included variables were examined (Table 2), mostly confirming the above-stated hypotheses showing small to large correlations. However, no significant associations were found between metabolic control and adolescent or caregiver Diabetes Family Responsibility Questionnaire scores, or between metabolic control and gender, adherence and gender regardless of respondent, or caregivers’ perception of adherence and caregiver education. In addition, caregiver perception of adherence was unrelated to their perception of supportive behavior in relation to diabetes care.

**Table 2:** Intercorrelations between health outcomes, psychosocial variables, and patient characteristics.

	HbA1c	ADQ (A)	ADQ (C)	BDI (A)	BAI (A)	SDQ (C)	SEDM (A)	FAD (A)	FAD (C)	DFCS (A)	DFCS (C)	DFBC sup. (A)	DFBC sup. (C)	DFBC non-sup. (A)	DFBC non-sup. (C)	DFRQ (A)	DFRQ (C)	Age	Gender	Diabetes duration (years)	Care-giver education level ^a^	Family structure	Insulin pump
HbA1c	1.00	-0.37**	-0.41**	0.17**	0.09*	0.25**	-0.27**	0.16**	0.19**	0.28**	0.35**	-0.10**	-0.13**	0.24**	0.24**	0.04	0.03	0.19**	-0.02	0.19**	-0.09*	0.13**	-0.12**
ADQ (A)		1.00	0.61**	-0.24**	-0.14**	-0.38**	0.62**	-0.38**	-0.31**	-0.37**	-0.39**	0.35**	0.23**	-0.29**	-0.32**	0.11**	-0.02	-0.27**	-0.07	-0.17**	0.11*	-0.13**	0.17**
ADQ (C)			1.00	-0.23**	-0.14**	-0.48**	0.52**	-0.27**	-0.39**	-0.40**	-0.55**	0.15**	0.08	-0.37**	-0.51**	0.15**	0.18**	-0.15**	-0.06	-0.15**	0.04	-0.16**	0.23**
BDI (A)				1.00	0.84**	0.44**	-0.37**	0.37**	0.22**	0.37**	0.18**	-0.15**	0.03	0.27**	0.18**	0.00	0.05	0.08*	-0.22**	0.00	-0.11**	0.08	-0.02
BAI (A)					1.00	0.38**	-0.33**	0.32**	0.16**	0.34**	0.12**	-0.12**	0.08	0.21**	0.13**	-0.02	0.02	0.04	-0.23**	-0.04	-0.10*	0.05	-0.02
SDQ (C)						1.00	-0.38**	0.35**	0.42**	0.35**	0.43**	-0.08	0.05	0.31**	0.40**	-0.19**	-0.30**	-0.03	0.13**	0.06	-0.14**	0.13**	-0.19**
SEDM (A)							1.00	-0.39**	-0.29**	-0.40**	-0.34**	0.22**	0.04	-0.32**	-0.32**	0.31**	0.21**	-0.00	0.03	-0.05	0.11**	-0.12**	0.09*
FAD (A)								1.00	0.47**	0.29**	0.28**	-0.33**	-0.15**	0.31**	0.22**	-0.03	-0.09*	0.13**	0.03	0.04	-0.10*	0.11**	-0.03
FAD (C)									1.00	0.26**	0.29**	-0.11**	-0.11**	0.26**	0.32**	-0.07	-0.13**	0.05	0.07	0.12**	-0.14**	0.05	-0.14**
DFCS (A)										1.00	0.45**	-0.04	0.01	0.49**	0.34**	-0.21**	-0.12**	0.05	-0.07	0.06	-0.09*	0.07	0.18**
DFCS (C)											1.00	-0.04	0.12**	0.37**	0.65**	-0.21**	-0.27**	0.01	0.12**	0.11**	-0.09*	0.04	-0.07
DFBC sup. (A)												1.00	0.45**	0.09*	0.05	-0.23**	-0.25**	-0.34**	0.08	-0.10*	-0.01	-0.01	0.04
DFBC sup. (C)													1.00	0.07	0.19**	-0.31**	-0.37**	-0.40**	0.01	-0.12**	-0.03	-0.01	0.11**
DFBC non-sup. (A)														1.00	0.53**	-0.08*	-0.20**	0.03	0.06	0.06	-0.07	0.03	-0.07
DFBC non-sup. (C)															1.00	-0.16**	-0.26**	-0.07	0.09*	0.05	-0.07	-0.11**	-0.08*
DFRQ (A)																1.00	0.57**	0.48**	-0.13**	0.05	-0.06	-0.09*	-0.04
DFRQ (C)																	1.00	0.51**	-0.17**	0.10*	0.02	-0.07	-0.05
Age																		1.00	0.02	0.19**	-0.05	0.02	-0.15**
Gender																			-1.00	0.03	0.01	0.03	-0.16**
Diabetes duration (years)																				1.00	0.01	-0.02	0.16**
Care-giver education level^a^																					1.00	-0.04	0.03
Family structure																						1.00	-0.07
Insulin pump																							1.00

HbA1c, hemoglobin A1c; ADQ, Adherence in Diabetes Questionnaire; BDI, Depression subscale of the Beck’s Youth Inventories–Second edition; BAI, Anxiety subscale of the Beck’s Youth Inventories–Second edition; SDQ, Strength and Difficulties Questionnaire; SEDM, Self-efficacy for Diabetes Self-Management; FAD, Family Assessment Device; DFCS, Diabetes Family Conflict Scale; DFBC sup., Diabetes Family Behavior Checklist–caregiver support subscale; DFBC non-sup., Diabetes Family Behavior Checklist–caregiver non-support subscale; DFRQ, Diabetes Family Responsibility Questionnaire. (A) indicates adolescent self-report, (C) indicates caregiver report.

^a^ Higher scores indicate higher levels of education.

**p < 0.01

*p < 0.05

#### Parent-adolescent correlations

Significant correlations were found between caregiver and adolescent reports for all measures, indicating relatively high agreement between the respondents. Large correlations were found for measures of adherence (*r* = 0.61), parental diabetes-related non-support (*r* = 0.53), and diabetes family responsibility (*r* = 0.57). Medium correlations were found for general family functioning (*r* = 0.47), diabetes-related family conflict (*r* = 0.45), and parental diabetes-related support (*r* = 0.45). In addition, medium correlations were found between parental Strength and Difficulties Questionnaire reports and adolescents’ self-reports on the Beck’s Depression Inventory (*r* = 0.44) and Beck’s Anxiety Inventory (*r* = 0.38).

### Path modeling

Two structural equation models of associations between adherence and metabolic control and diabetes-related family conflict, supportive and non-supportive caregiver behavior, responsibility for diabetes-related tasks, general family functioning, self-efficacy (only in the adolescent model), and social and emotional difficulties of the adolescent were tested, including the possible mediational role of adherence. Demographic variables were included as covariates.

#### Adolescent path model

**Fig 1:**
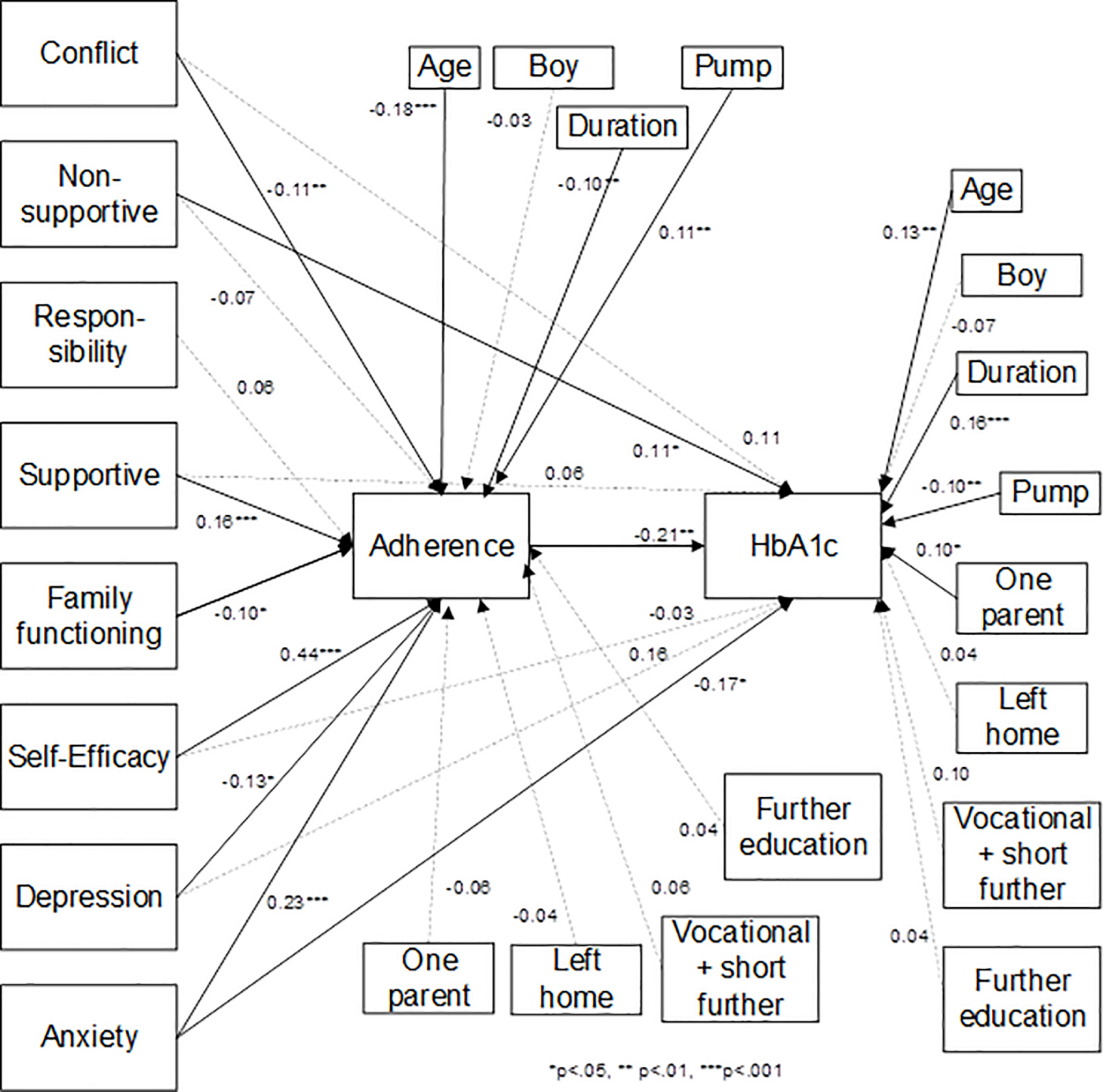
Standardized path coefficients–adolescent path model.

For adolescents, the model fit was satisfactory [*X*^2^(2) = 3.19, p = 0.20, CFI = 0.997, TLI = 0.955, SRMR = 0.004, RMSEA = 0.034] (Fig 1).

Higher non-supportive caregiver behavior (β = 0.11, p = 0.031), fewer symptoms of anxiety (β = -0.17, p = 0.017), and poorer adherence (β = -0.21, p = 0.001) were associated with poorer metabolic control. Less diabetes-related conflict (β = -0.11, p = 0.007), more supportive caregiver behavior (β = 0.16, p = 0.000), higher self-efficacy (β = 0.44, p = 0.000), lower family functioning (less unhealthy functioning) (β = -0.10, p = 0.011), fewer depressive symptoms (β = -0.13, p = 0.038), and more symptoms of anxiety (β = 0.23, p = 0.000) were associated with better adherence.

Longer diabetes duration (β = 0.16, p = 0.000), use of multiple daily injections vs. insulin pump (β = -0.10, p = 0.006), and living alone with one parent vs. living with both parents (β = 0.10, p = 0.030) were associated with poorer metabolic control. Lower age (β = -0.18, p = 0.000), shorter diabetes duration (β = -0.10, p = 0.003), and using an insulin pump vs. multiple daily injections (β = 0.11, p = 0.000) were associated with better adherence.

Finally, adherence fully mediated the relationship between metabolic control and diabetes-related family conflict, supportive caregiver behavior, and self-efficacy with bias-corrected bootstrapped confidence intervals (CIs) of [0.000; 0.043], [-0.057; -0.009], and [-0.149; -0.033], respectively. Adherence acted as a partial mediator for the relation between anxiety and metabolic control (CI [-0.084; -0.010]).

Overall, the proposed adolescent path model tested here accounted for 25% of the variance in metabolic control and 51% of the variance in adherence. Metabolic control had the strongest association with adherence (*r* = -0.21), and symptoms of anxiety (*r* = -0.17), whereas the variables most strongly associated with adherence were self-efficacy (*r* = 0.44) and symptoms of anxiety (*r* = 0.23)

#### Caregiver path model

**Fig 2:**
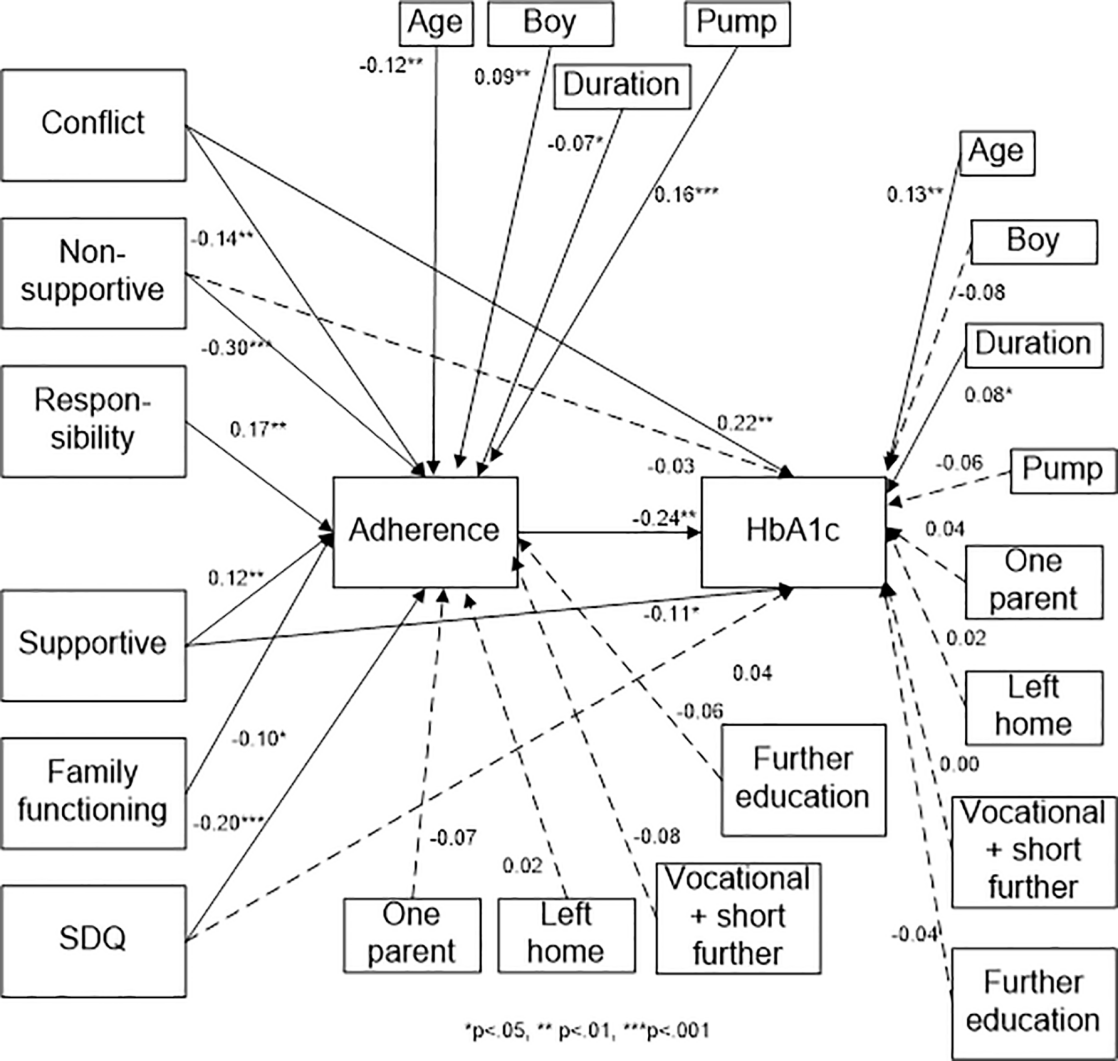
Standardized path coefficients–caregiver path model.

For caregivers, the model fit was satisfactory [*X*^2^(2) = 1.78, p = 0.41, CFI = 1.000, TLI = 1.008, SRMR = 0.003, RMSEA = 0.000] (Fig 2).

Higher diabetes-related conflict (β = 0.22, p = 0.001), less supportive caregiver behavior (β = -0.11, p = 0.013), and poorer adherence (β = -0.24, p = 0.001) were associated with poorer metabolic control. Less diabetes-related conflict (β = -0.14, p = 0.003), less non-supportive caregiver behavior (β = -0.30, p = 0.000), higher responsibility for diabetes-related tasks placed on the youth (β = 0.17, p = 0.006), more supportive caregiver behavior (β = 0.12, p = 0.001), and fewer social and emotional difficulties (β = -0.20, p = 0.000) were associated with better adherence. The higher the age of the adolescent (β = 0.13, p = 0.003) and the longer the duration of diabetes (β = 0.08, p = 0.027), the poorer the metabolic control, but lower age (β = -0.12, p = 0.002), being male (β = 0.09, p = 0.009), shorter duration of diabetes (β = -0.07, p = 0.022), and using an insulin pump vs. multiple daily injections (β = 0.16, p = 0.000) were associated with better adherence.

Finally, adherence fully mediated the relationship between metabolic control and lower non-supportive caregiver behavior and social and emotional difficulties (CIs: [0.021; 0.121] and [0.011; 0.084], respectively). Adherence partially mediated the relationship between metabolic control and diabetes-related family conflict, as well as supportive caregiver behavior (CIs: [0.004; 0.064] and [0.054; 0.002], respectively). With a slightly better model fit for the caregiver model than the adolescent model, the explanatory value regarding explained variances revealed substantial resemblance between the two models, with the caregiver path model accounting for 26% of the variance in HbA_1c_ and 49% of the variance in adherence. The variables most strongly associated with metabolic control were adherence (*r* = -0.24) and diabetes-related family conflict (*r* = 0.22), whereas adherence was most associated with parental non-support (*r* = -0.30) and the total Strength and Difficulties Questionnaire score (*r* = -0.20), reflecting the parental assessment of the social and emotional difficulties of the adolescent.

## Discussion

The present study investigated associations between patient characteristics, treatment aspects, and psychosocial and psychological variables on diabetes management and metabolic control. The associations between variables were assessed in two separate path models, one for adolescents and one for caregivers, and both had satisfactory fit.

The strengths of this study include the large and nationally representative sample size, multiple informants (adolescents and parents), and measures that included multiple methods (self-reports, register based, and biological). The response rate of this study was also relatively high compared to previous questionnaire-based studies.

The hypotheses tested here were derived from previous research based primarily on bivariate associations between a limited number of variables. By replicating this type of bivariate statistical procedure, our data confirmed the majority of these hypotheses. However, when integrating the same variables in adolescent and caregiver path models, the nature, and significance of some of these associations changed or disappeared, rejecting some of the hypotheses. Self-efficacy, emotional well-being of the adolescent, and difficulties in relation to adolescent/caregiver interactions (parental non-support and family conflict) proved to be particularly important aspects for understanding individual variability in adherence and metabolic control among adolescents with T1DM.

### Family-related factors

Both adolescent and caregiver assessment of diabetes-related parental support were related to adherence, whereas only caregiver assessment was related directly to metabolic control. Moreover, caregiver appraisal of adolescent adherence behavior partially mediated the relationship between parental assessment of support and metabolic control. Previous studies have supported an association between parental assessment of family support and metabolic control [31]. Parents’ perception of support could be speculated to be a reflection of a positive family milieu and a parenting style that increases the social and diabetes-related competencies of the adolescent, potentially decreasing the stress associated with living with T1DM in some adolescents. Other studies have suggested that worsening metabolic control, could lead parents to withdraw their support [32].

The adolescent model concurs with that of Lewin et al. [29], who found reports of parental support by children to be associated with adherence but not HbA_1c_. However, the relationship between adolescent assessment of parental support and adherence in the current study was relatively weak. Previous studies suggest that, in adolescents, it may be beneficial to consider the influence of both caregivers’ and friends’ support in predicting better adjustment to living with a chronic illness [59, 60].

Adolescent assessment of diabetes-related parental non-support was related to metabolic control but not to adherence. The reverse was found for the caregiver assessments; non-support was related to adherence but not to metabolic control. Perhaps adolescents’ perception of parental non-supportive behavior, such as critical and negative parenting, contributes to a stressful environment for the youth with diabetes which might affect their HbA_1c_ level, regardless of their adherence efforts. High levels of stress have previously been linked to poor metabolic control [61]. Ott et al. [25] found that parental non-support as rated by adolescents was related to blood glucose monitoring but not to other components of adherence. We did not assess the association between individual aspects of adherence and metabolic control, which may have altered the result regarding the lack of association between non-support and adherence. Moreover, it may be the lack of supportive behavior, such as positive encouragement and affirmation, more than adolescent perception of parental non-support that interacts with adolescents’ adherence behavior. Criticizing and nagging behaviors by their caregivers may influence other aspects of the daily lives of adolescents with T1DM. Caregiver reports in the current study indicate that caregivers who perceive their actions towards their youngsters as more non-supportive, such as criticizing and nagging, also perceive their adolescent as less adherent to diabetes care. Perhaps, caregivers who feel prone to criticizing are more focused on any sign of what they perceive as non-adherent behavior in their child or are more reactive when it comes to non-adherence.

In the adolescent model, no direct association between diabetes-related family conflict and metabolic control was found, challenging a number of previous findings based on either adolescent report or merged adolescent-caregiver conflict scores [26, 28]. Instead, mediation analysis revealed that the relationship between HbA_1c_ and conflict was fully mediated by adherence, which is corroborated by previous studies applying similar statistical methods [29, 62]. A family climate characterized by high levels of conflict has been speculated to lead to adolescent stress, and that stress may affect metabolic control [28]. However, the adolescent-reported level of conflict in the current sample was significantly lower (*M =* 20.6) than the normative sample for the Diabetes Family Conflict Scale (*M* = 24.4) [26], which may indicate an equally low level of stress and no subsequent effect on metabolic control.

In contrast, in the caregiver model, higher levels of family conflict were associated with poorer HbA_1c_ levels. This result is more in concordance with Drotar et al. [28] and Hood et al. [26], who also found an association between diabetes-related conflict in the family (based on either an adolescent-caregiver merged conflict score, or caregiver scores, respectively) and metabolic control. However, in the caregiver path model, the relationship between conflict and HbA_1c_ levels was partially mediated by adherence, indicating that conflict as assessed by caregivers is both directly and indirectly associated with metabolic control, possibly due to the physiological stress caused by the presence of family conflict.

The hypothesized relationship between the division of diabetes-related responsibilities and adherence was not confirmed in the adolescent path model. Perhaps the result would have been different had we used the Diabetes Family Responsibility Questionnaire to assess parent-child *agreement/disagreement* regarding the taking of responsibility. This factor has previously been linked to adherence [36]. Helgeson et al. [63] used the Diabetes Family Responsibility Questionnaire to assess *sharing of diabetes-related responsibility* and found that parent and child sharing of responsibility was associated with self-management, whereas either parent or child taking responsibility was not. Thus, they also highlighted a possible problem in scoring the Diabetes Family Responsibility Questionnaire as a continuous scale ranging from child to caregiver responsibility, as was done in this study.

The caregiver model showed an association between caregiver assessment of responsibility and adherence, indicating that adolescents who the caregiver saw as taking more responsibility for diabetes care tasks were also perceived as exhibiting better adherence toward treatment. This finding is somewhat contradictory with previous studies, which found that higher levels of adolescent responsibility are associated with a decrease in self-management behavior [64]. One possible explanation could be that caregivers who transfer the majority of treatment responsibilities to their adolescents are confident, or at least hopeful, regarding their child’s ability to take on these tasks, which is then reflected in their assessment of adherence. Whether this is an accurate appraisal could be questioned, as it is not reflected in the adolescents’ responses.

For both adolescents’ and caregiver’s reports, good general family functioning was related to better adherence. This finding is in line with previous research indicating family functioning to be associated with adherence based on both adolescent and caregiver reports [38, 65] and also our results regarding caregiver support, which could be considered an aspect of a positive family milieu.

### Assessment of emotional health and diabetes-related self-efficacy

Better metabolic control was associated with increased anxiety, which contradicts the results of our bivariate analysis and the majority of previous studies [11]. However, some other studies have found that internalizing problems, or specifically anxiety, is related to improved metabolic control based on either adolescent or caregiver assessments [66, 67]. One possible explanation for this association could be that fear of hypoglycemia leads adolescents to strive for a higher blood sugar level, reducing the risk of hypoglycemia, and perhaps also their level of anxiety [68].

We could also speculate that a certain level of anxiety in adolescents with T1DM could be adaptive. We previously found that the overall level of anxiety symptoms in Danish children and adolescents with T1DM are comparable or lower than in a normative Danish sample, indicating that the mental health of this patient group is fairly good [13]; the current sample was part of that study and we therefore assume that, the level of anxiety is generally not at a critical or impairing level. Symptoms of anxiety or an anxiety-prone personality in some individuals with T1DM may lead to more adherence toward treatment recommendations to prevent or reduce the anxiety-inducing consequences of non-adherence and poor metabolic control, leading to better metabolic control. The adolescent path model also showed that, though adherence moderately correlated with anxiety, it also acted as a partial mediator in the association between anxiety and HbA_1c_.

Even though the bivariate correlation analysis showed a significant association between symptoms of depression and HbA_1c_, in the path model the relationship between these two variables became non-significant. This contradicts a number of previous studies in which depression was shown to be associated with metabolic control [12]. A possible explanation for our finding could be that symptoms of depression do not always have a direct effect on metabolic control, but lead instead to a decrease in diabetes self-management tasks, as evidenced by our analysis showing adherence as a partial mediator of the relationship between HbA_1c_ and symptoms of depression, which is also supported by previous findings based on adolescent assessment [29]. Symptoms of depression may also have an even more indirect effect on adapting to living with diabetes, which was not tested in this study, through effects on the general family milieu and functioning, child/adolescent self-efficacy, or an increase in the diabetes-related conflict in the family. In their review of anxiety and depression in juvenile diabetes, Dantzer et al. [19] highlighted how several studies have suggested that adaptation plays a more important role in predicting metabolic control than symptoms of depression, but that depression may influence this process of adaptation. Again, it is important to note that the general psychological well-being of Danish adolescents with T1DM in the presents study appears to be less impacted compared to other samples which might also have affected the results [11, 13]. More studies are needed to understand the complex interaction of diabetes and depression, not disregarding the complex contributions of physiological factors [69].

The association between caregiver assessments of the social and emotional health (the Strength and Difficulties Questionnaire) of adolescents and metabolic control was fully mediated by adherence. This result appears to support the lack of direct correlation between symptoms of depression and metabolic control found in the adolescent path model.

Regarding adolescent assessment of self-efficacy in relation to diabetes care, no direct association with metabolic control was found, contradicting a number of previous studies [21, 54]. Instead, the association was fully mediated by adherence, supporting the work of Herge et al. [70]. Self-efficacy has previously been found to be related to active coping behaviors, and it could be that it is these behaviors, more than self-efficacy, that affect metabolic control [71]. The association between adherence and self-efficacy (*r* = 0.44) was the strongest association found in the adolescent path model and highlights the importance of this aspect of adolescents’ adaption to living with T1DM.

### Demographic and treatment-related variables

Both the adolescent and caregiver path models showed that better adherence is associated with lower HbA_1c_ levels, just as longer duration of T1DM and older age of the adolescent is related to both decreased adherence level and worse metabolic control. Both the adolescent and caregiver models showed an association between CSII treatment and better adherence. However, only the adolescent model found CSII treatment to be related to improved metabolic control.

In contrast to the adolescent model, the caregivers’ report of the living situation of the adolescent was not associated with HbA_1c_. In addition, the education level of the caregiver was unrelated to adherence and metabolic control in both models. The gender of the adolescent was also not associated with metabolic control in either model, but weakly associated with adherence in the caregiver model, indicating that caregivers perceive boys with T1DM to be slightly more adherent than girls, replicating the conclusion of others [72].

Overall, the adolescent path model accounted for 25% of the variance in metabolic control and 51% of the variance in adherence, whereas the caregiver path model accounted for 26% of the variance in HbA_1c_ and 49% of the variance in adherence. Thus, our results regarding explained variance are comparable, or even an improvement on previous multivariate models [5].

Comparing the two models based on explained variance, no substantial differences were found. However, the caregiver model had a slightly better model fit. Based on findings regarding associations between included variables, this study highlights the additional information gained from consulting both adolescents and caregivers.

### Limitations

Our results should be interpreted in light of several limitations. First, although several risk factors for poor adherence and metabolic control are identified in this study, the cross-sectional design prevents us from offering causal or bidirectional explanations for the relationships between the included variables. In particular, as demonstrated by Maxwell and Cole [73], cross-sectional analyses of mediation may be biased and hence causality cannot be determined. Second, the fact that non-participants had less optimal metabolic control than adolescents from participating families may also have influenced the outcome, though this bias is commonly found in this type of research. We can only speculate that the inclusion of these participants may have strengthened the associations between the investigated variables and outcomes. Third, with only 1.3% of the participants reporting the preferred language spoken in the home not being Danish, an insufficient number of minority families participated in the study to determine whether the results may generalize to this group. Furthermore, 83.6% of the responding caregivers were female, with 98.5% being the biological mother of the adolescent. Previous studies have shown that fathers have a somewhat differing view of the treatment efforts and emotional well-being of the child with diabetes [74]. Fourth, the relatively low internal consistency found for both the supportive and non-supportive subscales of the Diabetes Family Behavior Checklist may have had effect on the results involving these subscales. The reliability of this questionnaire has previously been found to be somewhat low [75], and future research could benefit from a more reliable measure of family support to confirm the results of the current study.

Lastly, dividing participants into smaller age segments may have revealed different patterns of interactions between psychosocial variables and outcomes that did not appear in our models, as others have found age to act as a moderator of the family functioning-adherence-metabolic control associations [76], and that developmental needs and reliance on caregivers vary greatly throughout the course of adolescence [77].

Although one of the major strengths of this study was the large, national, demographically diverse sample, the relatively low level of poverty in the Danish community, the financial support, and free access to a public health care system that covers many of the medically related expenses of children and adolescents with T1DM distinguishes this population, to some degree, from many other national samples.

### Clinical implications

This study adds to the growing body of research highlighting the importance of addressing the psychological and psychosocial well-being of adolescents with T1DM.

As the relationship between adherence and self-efficacy was the strongest association in the adolescent model, this study emphasizes the importance of adolescent self-efficacy in relation to diabetes care. Finding a way to build or strengthen the adolescents’ beliefs in their ability to overcome obstacles in relation to their daily self-management of diabetes care may increase the attention given to the adherence behavior of this group, improving metabolic control.

This study agrees with previous results stressing the importance of monitoring the emotional well-being of adolescents with T1DM, as the presence of emotional difficulties could affect the adolescents’ adherence level. Interventions focusing on the importance of a supportive family milieu would probably prove beneficial, both with respect to adherence and metabolic control.

Diabetes duration may also be a focal point in the clinical care of children and adolescents, not only because of the physiological implications of a longer duration of T1DM, but also because adolescents with a longer duration have been shown to be in need of additional support to re-commit and improve diabetes care activities and goals [4].

Adolescent self-efficacy, finding a way to handle anxiety without damaging diabetes outcomes, and educating caregivers with regard to developmentally appropriate levels of support appear to be valuable focal points.

## Supporting information

S1 Fig Study inclusion. (PDF)
