# Supplementary figures and images for: Multi-informant path models of the influence of psychosocial and treatment-related variables on adherence and metabolic control in adolescents with type 1 diabetes mellitus
Source: PLoS One. 2018 Sep 20;13(9):e0204176. doi: 10.1371/journal.pone.0204176 (PMC6147740; doi:10.1371/journal.pone.0204176)

**S1 Fig. Study inclusion**

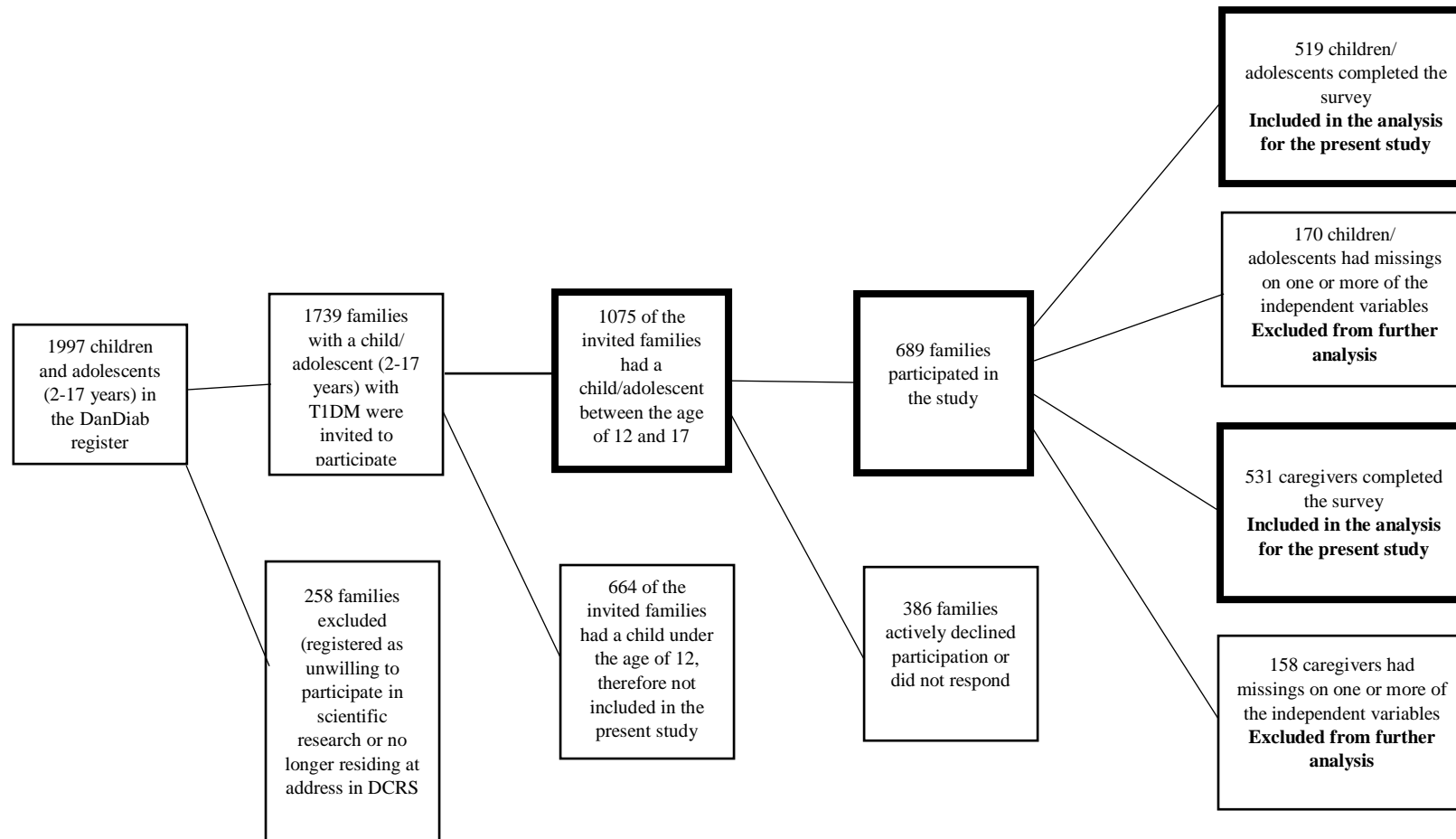

Supplement: S1 Fig — (PDF) [file pone.0204176.s001.pdf]
